# Emergency Laparoscopic Appendectomy in a High-Risk Cardiac Patient With Severe Left Main Coronary Artery Disease on Dual Antiplatelet Therapy

**DOI:** 10.14740/jmc5327

**Published:** 2026-07-01

**Authors:** Blerim Arapi, Alma Cani, Rudin Domi, Gentian Huti, Asead Abdyli, Krenar Lilaj, Filadelfo Coniglione, Mustafa Bajraktari, Rinard Kortoci, Majlinda Naco, Arjana Sina, Migena Vargu, Ergita Nelaj, Vedat Eljezi

**Affiliations:** a Department of Anesthesia and Intensive Care, Hygeia International Hospital, Tirana, Albania; b Department of Surgery, University of Medicine, Tirana, Albania; c Department of Anesthesia and Intensive Care, American Hospital 3, Tirana, Albania; d Department of Clinical Science and Translational Medicine, Tor Vergata University of Rome, Rome, Italy; e Department of Anesthesia and Intensive Care, American Hospital 2, Tirana, Albania; f Imaging Service, Wellness Albania Clinic, Tirana, Albania; g Department of Infectious Diseases, University of Medicine, Tirana, Albania; h Department of Internal Medicine, University of Medicine, Tirana, Albania; i Department of Perioperative Medicine, CHU Gabriel-Montpied, Clermont-Ferrand, France

**Keywords:** Anesthesia, Coronary stents, Coronary artery disease, Dual antiplatelet therapy, Emergent surgery

## Abstract

Emergency noncardiac surgery in patients with advanced coronary artery disease and recent coronary stent implantation poses a substantial anesthetic challenge. Clinicians must carefully balance the heightened risk of perioperative bleeding associated with continued dual antiplatelet therapy against the potentially catastrophic risk of stent thrombosis if such therapy is interrupted. We described the perioperative management of a 68-year-old man with severe multivessel coronary artery disease, critical left main stenosis, impaired left ventricular function, and recent coronary stenting, who required emergency laparoscopic appendectomy for perforated appendicitis. General anesthesia was administered with comprehensive invasive hemodynamic monitoring and tailored vasopressor support. The procedure was completed successfully, and the postoperative course was uneventful. This case underscored the feasibility of proceeding with emergency laparoscopic abdominal surgery without interruption of dual antiplatelet therapy in a patient with high-risk coronary anatomy, including left main disease. It highlighted a pragmatic, individualized anesthetic strategy integrating advanced hemodynamic monitoring, goal-directed support, and close multidisciplinary coordination, offering practical insight into managing the competing risks of bleeding and stent thrombosis in time-critical surgical settings.

## Introduction

Patients with advanced coronary artery disease (CAD) who require urgent noncardiac surgery represent a particularly high-risk population, facing significant challenges from both cardiac and bleeding complications [1]. The presence of recent coronary stent implantation further increases perioperative vulnerability. Interrupting dual antiplatelet therapy (DAPT) to reduce bleeding risk can precipitate acute stent thrombosis, which is often fatal, whereas continuing DAPT increases the likelihood of significant perioperative bleeding, particularly in intra-abdominal procedures [2, 3]. These patients are also highly susceptible to myocardial ischemia, arrhythmias, and hemodynamic instability during surgery due to impaired coronary perfusion, reduced ventricular function, and stress response associated with emergency procedures [4]. Emergent conditions such as perforated appendicitis compound these risks, leaving no opportunity to safely delay surgery.

Guidelines from major societies provide structured recommendations to navigate these complex scenarios. The American College of Cardiology (ACC) advises a comprehensive preoperative risk assessment that considers recent coronary interventions, stent type and timing, left ventricular function, and functional capacity. Elective surgery is generally deferred for at least 30 days after bare-metal stent placement and 6 months after drug-eluting stent implantation to reduce the risk of stent thrombosis. In urgent or emergent situations, ACC guidance emphasizes continuation of DAPT whenever feasible and close multidisciplinary coordination to balance the competing risks of bleeding and thrombosis [5].

Similarly, the American Heart Association (AHA) highlights individualized risk stratification and careful perioperative planning. Noninvasive testing, such as stress evaluation or imaging, may be appropriate in selected high-risk patients if time allows, but in emergencies, the AHA recommends maintaining DAPT whenever possible. Collaboration among cardiologists, anesthesiologists, and surgeons is crucial to optimize hemodynamic management, minimize bleeding risk, and prevent cardiac complications [6].

The European Society of Cardiology (ESC) provides detailed recommendations for patients with recent coronary stents undergoing noncardiac surgery. The ESC guidelines stress deferring elective procedures according to stent type, 30 days for bare-metal stents and 6 months for drug-eluting stents, and continuing DAPT in high-risk patients when possible. Perioperative risk stratification should consider coronary anatomy, ventricular function, and the urgency of surgery, with invasive hemodynamic monitoring and goal-directed therapy recommended for high-risk cases, particularly when emergent surgery cannot be delayed [7].

This report presents the successful anesthetic management of a 68-year-old patient with severe multivessel CAD, critical left main stenosis, recent coronary stenting, and ongoing DAPT who required emergency laparoscopic appendectomy for perforated appendicitis.

The case demonstrates that emergency minimally invasive abdominal surgery can be safely performed in patients with high-risk coronary anatomy without interrupting DAPT, a scenario that remains scarcely addressed in the current literature. Unlike most previous reports, which focus on elective surgeries or theoretical recommendations, this case illustrates the practical challenges of balancing bleeding and thrombotic risks in an urgent clinical setting. By applying a physiology-driven, multidisciplinary strategy that integrates established guideline recommendations with individualized perioperative care, this report may provide a useful clinical example for the management of urgent noncardiac surgery in patients with recent stents.

## Case Report

### Investigations

A 68-year-old man presented with a 4- to 5-day history of abdominal pain, initially localized to the right posterior abdomen and later migrating to the right iliac fossa, with associated localized guarding. Clinically, the patient was initially suspected of having appendicitis, and a routine abdominal ultrasound was performed as the first-line imaging modality. However, the definitive diagnosis was established by computed tomography (CT) scan, which confirmed acute appendicitis and accurately defined the stage of disease, including perforation and advanced inflammatory involvement. In this high-risk patient, CT imaging was essential not only for diagnostic confirmation but also for assessment of disease severity and for supporting the decision that urgent surgical intervention was necessary (Fig. 1). Preoperative evaluation revealed an extremely high-risk cardiovascular profile. The patient had known triple-vessel CAD, including approximately 85% left main coronary artery stenosis, complete right coronary artery occlusion with collateral retrograde flow from the left anterior descending artery, and severe (90%) left circumflex artery stenosis. One month earlier, he had undergone stenting of the left anterior descending and D1 arteries (Fig. 2) and remained on DAPT with prasugrel 10 mg and aspirin 100 mg daily. A prior circumflex stent had been placed 15 years earlier, and coronary artery bypass grafting was already planned.

**Figure 1:**
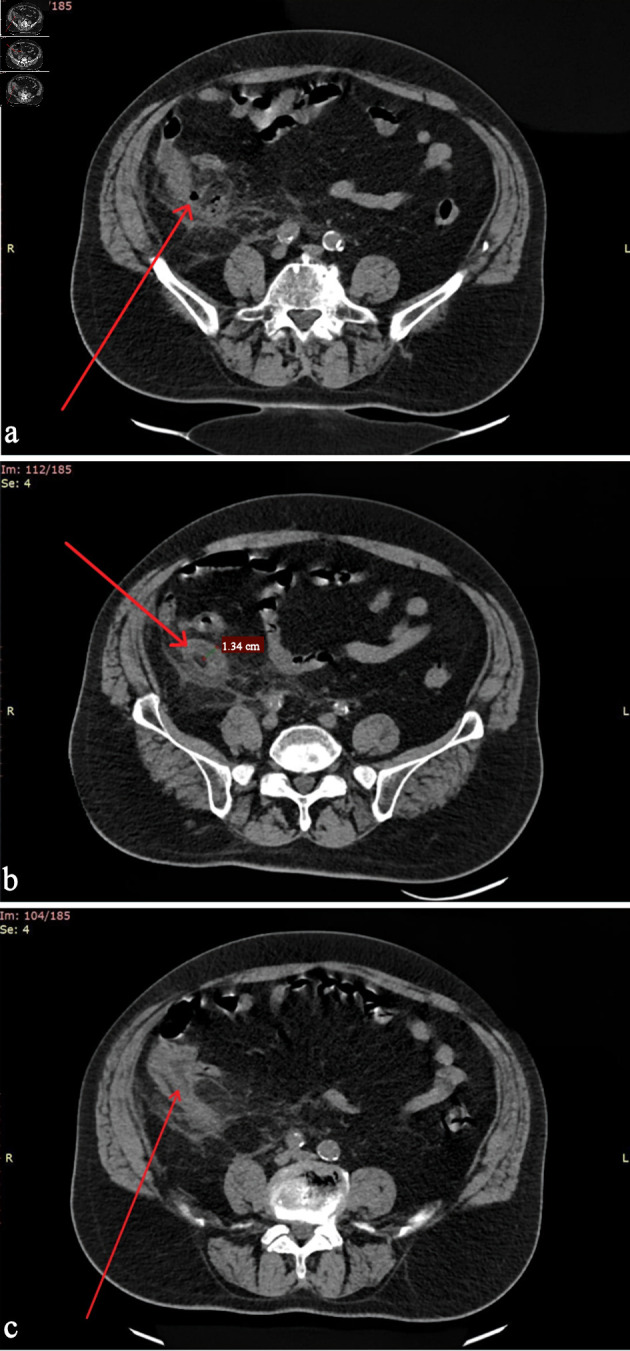
Abdominal CT scans demonstrating a perforated and severely inflamed appendix with appendiceal wall thickening and evidence of structural destruction (arrows). (a) Perforated appendix with structural destruction. (b) Marked appendiceal inflammation with increased wall thickness. (c) Additional axial view demonstrating perforation and inflammatory changes. CT: computed tomography.

**Figure 2:**
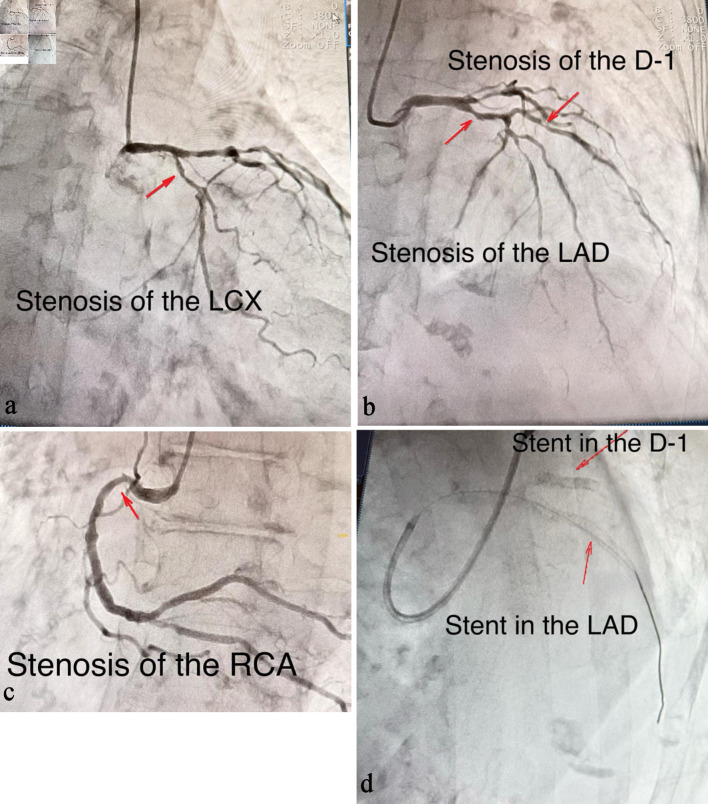
Coronary angiography and post-intervention findings. (a) Stenosis of the left circumflex artery (LCX). (b) Stenosis of the left anterior descending (LAD) and first diagonal (D-1) arteries. (c) Stenosis of the right coronary artery (RCA). (d) Final angiographic result after percutaneous coronary intervention and balloon angioplasty demonstrating stent placement in the LAD and D-1 arteries.

Preoperative transthoracic echocardiography demonstrated severe left ventricular dysfunction with an ejection fraction of 30%, moderate mitral regurgitation, and marked left ventricular dilation (end-diastolic diameter 58 mm), consistent with advanced chronic heart failure; previously documented ejection fraction values ranged between 35% and 40%. Additional comorbidities included insulin-treated diabetes mellitus and arterial hypertension treated with bisoprolol and valsartan, while furosemide 25 mg daily was prescribed for heart failure management.

Objective perioperative risk stratification further confirmed the patient’s critical status. The Revised Cardiac Risk Index (RCRI) was 3 points and American Society of Anesthesiologists (ASA) Physical Status IV-E, based on ischemic heart disease, congestive heart failure, and insulin-treated diabetes mellitus, corresponding to a markedly increased risk of major perioperative cardiac complications during emergency surgery.

### Diagnosis

The patient was diagnosed with perforated appendicitis with localized peritonitis, representing a contained intra-abdominal infection. The patient’s diagnosis was particularly challenging due to the coexistence of advanced cardiovascular disease, recent coronary stenting, and ongoing DAPT, which markedly increased the risk of perioperative complications, including hemodynamic instability, myocardial ischemia, and bleeding. This combination of surgical urgency and high cardiac risk underscored the need for careful planning and expedited operative intervention.

### Treatment

Although current guidelines generally recommend delaying noncardiac surgery for 3–6 months following drug-eluting stent implantation, our patient required urgent surgical intervention because of perforated appendicitis, making further postponement clinically unsafe. Delaying surgery would have exposed the patient to substantial risks related to ongoing intra-abdominal infection and sepsis.

Emergency laparoscopic appendicectomy was undertaken given the risk of progression to generalized peritonitis and sepsis. The patient was transferred to the operating theatre, where standard monitoring was applied. Invasive arterial blood pressure monitoring was established prior to induction of anesthesia (Fig. 3). Comprehensive monitoring in accordance with ASA guidelines was instituted, incorporating transesophageal echocardiography (TEE) to evaluate cardiac function and optimize fluid therapy (Figs. 4, 5). General anesthesia was delivered with comprehensive invasive hemodynamic monitoring and carefully titrated vasopressor support, allowing safe completion of the procedure and an uneventful postoperative recovery.

**Figure 3:**
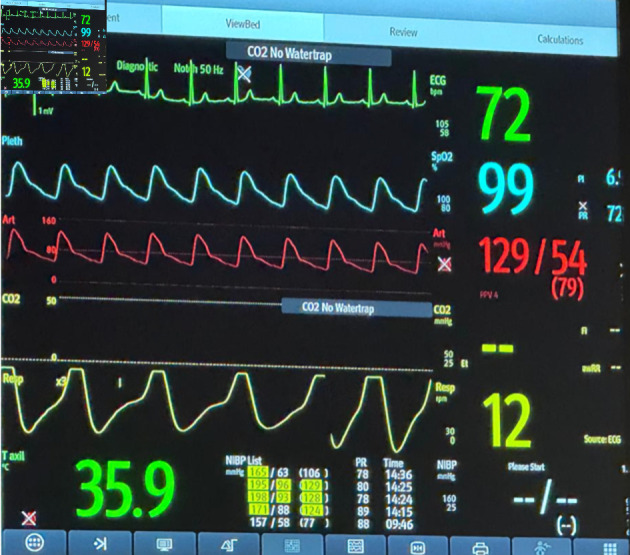
Hemodynamic intraoperative monitoring.

**Figure 4:**
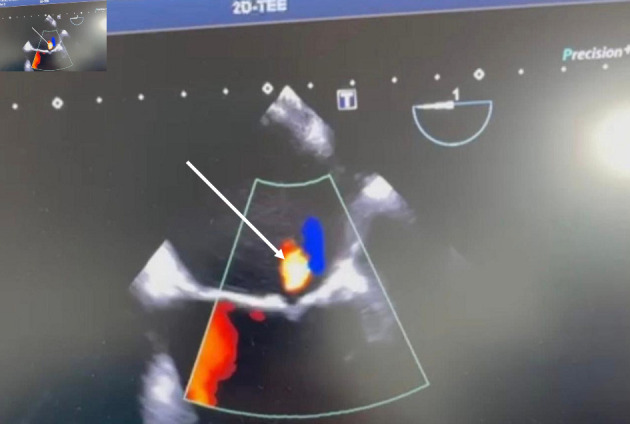
Intraoperative transesophageal echocardiography showing mitral regurgitation; color Doppler demonstrates systolic flow from the left ventricle to the left atrium (white arrow).

**Figure 5:**
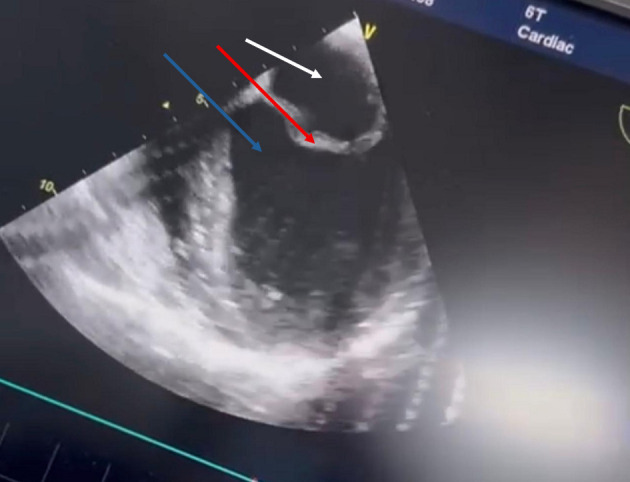
Intraoperative transesophageal echocardiography of the left atrium and ventricle for functional assessment and fluid guidance (left atrium: white arrow; mitral valve: red arrow; left ventricle: blue arrow).

General anesthesia was induced and maintained with a remifentanil infusion and sevoflurane. A central venous catheter was inserted following induction to facilitate hemodynamic monitoring and administration of vasoactive agents.

Intraoperatively, hemodynamic support with a norepinephrine 0.05–0.1 µg/kg/min infusion was required to maintain adequate arterial pressure and coronary perfusion. The patient was mechanically ventilated using a lung-protective strategy (tidal volume 6 mL/kg, plateau pressure < 30 cm H_2_O, and driving pressure < 13 cm H_2_O), with a low level of positive end-expiratory pressure (5 cm H_2_O). A laparoscopic appendectomy was performed with the patient in approximately 30° Trendelenburg position, using a low pneumoperitoneum pressure (8–10 mm Hg) to minimize impairment of venous return, preload, and renal perfusion. Intraoperative findings revealed a retrocecal, perforated appendix. Careful dissection was undertaken, the appendix was removed, and the stump secured. Extensive peritoneal lavage was performed. The total operative time was approximately 3 h. Table 1 summarizes the main perioperative anesthetic and hemodynamic parameters, including anesthetic agents, intraoperative mean arterial pressure targets, blood loss estimation, perioperative hemoglobin trend, and postoperative cardiac evaluation.

**Table 1:** Perioperative Anesthetic, Hemodynamic, and Postoperative Cardiac Assessment Parameters

Parameter	Details
Induction agents	Propofol 150 mg, fentanyl 200 µg, vecuronium 10 mg
Intraoperative MAP target	Maintained between 70–75 mm Hg
Estimated blood loss	No significant blood loss during the procedure
Hemoglobin trend	Preoperative: 12.3 g/dL; postoperative: 11.8 g/dL
Postoperative troponin I	0.008 µg/L (normal range 0–0.019)
Postoperative CK-MB	0.60 ng/mL (normal range < 6.22)
Postoperative ECG	No ischemic changes/evidence of myocardial ischemia

MAP: mean arterial pressure; CK-MB: creatine kinase–MB; ECG: electrocardiogram.

### Follow-up and outcomes

At the end of the procedure, the patient was carefully extubated and transferred to the intensive care unit for close monitoring. Hemodynamic parameters remained stable and drain output showed no evidence of postoperative bleeding. Although postoperative myocardial injury after noncardiac surgery (MINS) is an important perioperative consideration, postoperative troponin levels, electrocardiogram (ECG) evaluation, and transthoracic echocardiography examination in our patient showed no evidence of myocardial injury, and no changes on kinetics and ejection fraction compared to preoperative evaluation. DAPT with prasugrel and aspirin was continued throughout the perioperative period without interruption, and the first postoperative doses were administered on postoperative day 1. No anticoagulation was administered. Intravenous antibiotics were given for 5 days to treat peritonitis. The postoperative course was uneventful. The patient was discharged on the fifth postoperative day. Follow-up at 1 week was unremarkable, and he remained in good clinical condition at 1 month.

## Discussion

### CAD and coronary stents in the perioperative period: surgical timing and DAPT management

Emergency noncardiac surgery in patients with advanced CAD and recent coronary stent implantation poses a considerable clinical challenge, as it requires balancing the risks of perioperative bleeding with those of stent thrombosis. Interruption of DAPT is a well-established trigger for acute stent thrombosis, a complication associated with significant morbidity and mortality, particularly in patients with high-risk coronary anatomy, including left main disease, multivessel involvement, and impaired left ventricular function. Conversely, continuation of DAPT increases the risk of surgical bleeding, necessitating meticulous perioperative planning and mitigation strategies.

Current guidelines generally recommend delaying elective noncardiac surgery until after the critical post-stent period, at least 1 month for bare-metal stents and 3–6 months for drug-eluting stents [5–7]. However, these recommendations may be often impractical in urgent or emergent scenarios, underscoring the limitations of guideline-based approaches and highlighting the need for individualized decision-making.

Although current guidelines recommend delaying noncardiac surgery after drug-eluting stent implantation, several studies suggest that urgent surgery may be performed safely in carefully selected patients when delay is not feasible [8–10]. In our case, acute perforated appendicitis precluded postponement, requiring an individualized multidisciplinary strategy balancing thrombotic and bleeding risks.

Premature discontinuation of DAPT remains the strongest predictor of stent thrombosis, particularly within the first weeks to months after PCI, and is associated with high mortality [11–14]. Although our patient had not yet received the scheduled antiplatelet doses on the day of surgery, we deliberately chose to continue DAPT because of the extremely high thrombotic risk related to recent stenting and critical left main disease. This decision aligns with evidence suggesting that continuation of antiplatelet therapy may reduce ischemic complications without substantially increasing bleeding in urgent surgery [15–18].

To mitigate perioperative risk, a minimally invasive laparoscopic approach with low pneumoperitoneum pressures was selected, combined with comprehensive invasive hemodynamic monitoring and TEE, allowing continuous assessment of cardiovascular function throughout the procedure [19]. Table 2 [8–12, 14–23] summarizes the key findings of relevant studies and their relevance to our case. Our patient presented with acute perforated appendicitis, making surgical delay impossible. Management closely followed established perioperative principles for patients with coronary stents. Although discontinuation of DAPT was technically possible because the patient had not yet received the scheduled doses on the day of surgery, the decision was made to continue both agents perioperatively in view of the extremely high risk of catastrophic stent thrombosis. A minimally invasive laparoscopic approach was chosen to limit surgical stress and promote faster recovery, while low pneumoperitoneum pressures were used to minimize adverse effects on venous return, preload, and end-organ perfusion. Comprehensive invasive hemodynamic monitoring, complemented by TEE, enabled continuous assessment and optimization of blood pressure, heart rate, ventricular function, and tissue perfusion, ensuring strict cardiovascular stability throughout the perioperative period.

**Table 2:** Summary of Key Studies on Perioperative Management After Coronary Stenting and Their Relevance to Our Case

Study	Main findings	Relevance to our case
Lee et al, 2023 [8]	In 186 patients, early surgery (< 6 months after DES) was not associated with increased postoperative myocardial injury when preoperative hs-cTnI was normal.	Supports the feasibility of urgent surgery in selected high-risk patients when delay is not possible.
Xu et al, 2025 [9]	In 2,501 patients undergoing gastrointestinal cancer surgery after PCI, earlier surgery carried higher MACE risk, especially within the first 87 days.	Reinforces that delaying surgery is preferable, but urgent surgery may still be necessary in critical conditions.
Choi et al, 2010 [10]	Surgery within the first 3 months after DES implantation was associated with higher adverse outcomes.	Highlights the elevated perioperative risk in our patient with recent PCI.
Sharma et al, 2004 [11]	Early surgery within 90 days, especially < 3 weeks, had high mortality when thienopyridines were discontinued; continuation of antiplatelet therapy reduced mortality without increasing bleeding.	Supports our decision to continue DAPT perioperatively despite bleeding concerns.
Van Kuijk et al, 2009 [21]	Perioperative MACEs decreased as the interval between stenting and surgery increased; continued DAPT increased bleeding risk but did not fully prevent MACEs.	Demonstrates the complex balance between thrombosis and bleeding risk.
Damazo-Escobedo et al, 2022 [15]	Prolonged DAPT was associated with low major bleeding (1.5%) and mortality (3.7%), with low thrombosis/restenosis rates.	Suggests that continued DAPT may be tolerated without major bleeding excess.
Rossini et al, 2018 [20]	Emphasized multidisciplinary perioperative antithrombotic management tailored to surgical and thrombotic risk.	Reflects the multidisciplinary individualized strategy used in our patient.
Barash et al, 2010 [22]	Premature DAPT interruption increases stent thrombosis risk; urgent surgery requires individualized antiplatelet management.	Supports prioritizing thrombosis prevention in very high-risk coronary anatomy.
Gurajala et al, 2016 [12]	Urgent noncardiac surgery after coronary stenting requires individualized perioperative planning and monitoring.	Supports the tailored anesthetic and monitoring strategy applied in our case.
Vicenzi et al, 2006 [16]	Surgery soon after PCI, especially within 6 weeks, was associated with increased perioperative cardiac events.	Highlights the high-risk timing of surgery in our patient.
Faloye et al, 2025 [17]	Continuation of aspirin reduces stent thrombosis and ischemic events despite modest bleeding risk.	Supports maintaining antiplatelet therapy perioperatively.
Rohatgi et al, 2022 [18]	Aspirin continuation is generally recommended in urgent surgery, with individualized P2Y12 inhibitor management.	Relevant to perioperative DAPT decision-making in our patient.
Yoon et al, 2020 [14]	DAPT continuation is protective against 30-day MACEs; anesthetic technique itself does not independently affect outcomes.	Supports the importance of maintaining DAPT and intensive perioperative management.
Brilakis et al, 2007 [19]	Premature DAPT discontinuation is the strongest predictor of stent thrombosis and carries high mortality.	One of the main reasons DAPT was intentionally continued in our patient.
Watkin et al, 2023 [23]	Laparoscopy reduces surgical stress, but pneumoperitoneum can affect cardiovascular physiology, requiring careful monitoring.	Supports our use of low-pressure pneumoperitoneum, invasive monitoring, and TEE.

DES: drug-eluting stent; hs-cTnI: high-sensitivity cardiac troponin I; PCI: percutaneous coronary intervention; MACE: major adverse cardiovascular event; DAPT: dual antiplatelet therapy TEE: transesophageal echocardiography;

The decision to maintain DAPT was primarily guided by the patient’s very high thrombotic risk, including recent stent implantation and critical left main coronary stenosis, where the consequences of stent thrombosis were considered greater than the risk of surgical bleeding, as recent literature recommended [20, 21]. In addition, the laparoscopic approach helped mitigate hemorrhagic risk by reducing tissue trauma and intraoperative blood loss despite ongoing antiplatelet therapy.

### Anesthetic considerations in perioperative management

Morgan et al [24] outline key anesthetic principles for ischemic heart disease patients undergoing noncardiac surgery, emphasizing maintenance of myocardial oxygen supply–demand balance. Management should avoid tachycardia, hypotension, and hypertension, which can precipitate ischemia. Both volatile agents and propofol-based techniques are acceptable, with emphasis on hemodynamic stability and stress response control. Individualized anesthetic plans, appropriate invasive monitoring, and meticulous fluid and vasopressor management are recommended. Continuation of beta-blockers and guideline-directed therapy, alongside adequate analgesia, is advised to minimize sympathetic activation [22–25].

Lung-protective ventilation strategies were applied throughout the procedure, including the use of low tidal volumes, plateau pressures below 30 cm H_2_O, and driving pressures below 13 cm H_2_O. This approach aimed to minimize ventilator-induced lung injury while maintaining adequate oxygenation and respiratory mechanics during laparoscopic surgery [26].

Multidisciplinary collaboration was central to the favorable outcome, enabling coordinated risk assessment and perioperative planning. Table 3 summarizes key anesthetic and preoperative concepts.

**Table 3:** Key Anesthetic Recommendations for Patients With Coronary Stents Undergoing Noncardiac Surgery

Category	Recommendation/key point	Rationale/notes
Antiplatelet therapy	Continue DAPT, especially aspirin, when possible	Reduces risk of stent thrombosis; bleeding risk must be monitored
Anesthetic technique	Both volatile agents and total intravenous anesthesia (TIVA) acceptable	No clear evidence favors either; focus on hemodynamic stability
Hemodynamic management	Avoid tachycardia, hypotension, and abrupt hypertension	Prevents perioperative myocardial ischemia
Ventilation	Apply lung-protective ventilation (low tidal volume, plateau < 30 cm H_2_O, driving pressure < 13 cm H_2_O)	Reduces ventilator-induced lung injury and preserves oxygenation
Surgical approach	Prefer minimally invasive/laparoscopic surgery with low insufflation pressure	Minimizes hemodynamic compromise, tissue trauma, and blood loss
Monitoring	Use invasive hemodynamic monitoring (arterial line, continuous perfusion assessment)	Allows real-time adjustment of anesthetic depth, fluids, and vasoactive support
Fluid and vasopressor management	Tailor fluids and vasoactive drugs to maintain coronary perfusion	Maintains myocardial oxygen supply–demand balance
Postoperative care	Continue DAPT, monitor hemodynamics, assess for bleeding and cardiac complications	Ensures safe recovery and early detection of complications

DAPT: dual antiplatelet therapy.

Intraoperative management in our case adhered to these principles, maintaining myocardial oxygen balance, avoiding hemodynamic swings, applying lung-protective ventilation, low pneumoperitoneum pressures, and comprehensive invasive monitoring. Real-time adjustments of anesthetic depth, fluids, and vasoactive agents allowed precise cardiovascular control and minimized myocardial stress, consistent with literature recommendations for high-risk cardiac patients [22–26].

### Postoperative management and monitoring in high-risk cardiac patients

Postoperatively, vigilant surveillance for bleeding and cardiac complications was essential. Continuation of DAPT and close hemodynamic monitoring contributed to an uneventful recovery, though these results cannot be generalized.

Sellers et al emphasize that cardiovascular complications are major contributors to postoperative morbidity and mortality in patients with prior coronary stents, including MINS, myocardial infarction, arrhythmias, and heart failure. Many ischemic events are clinically silent, and stent thrombosis remains a rare but often fatal complication associated with premature DAPT discontinuation and the prothrombotic surgical milieu [27].

Postoperative care should be structured, clinically guided, and focused on cardiovascular risk reduction and early complication detection. Our patient, a 68-year-old man with severe multivessel CAD, critical left main stenosis, recent stenting, and ongoing DAPT, required careful attention to ischemic symptoms, ECG changes, and hemodynamic stability. Hypotension, tachycardia, anemia, and hypoxemia were actively avoided. Antiplatelet therapy was resumed as early as safely feasible, balancing bleeding and thrombotic risks. Follow-up prioritized clinical assessment, functional status, and risk factor management, with additional testing reserved for suspected ischemia. This approach underscores the importance of vigilant, symptom-guided monitoring and multidisciplinary coordination in optimizing postoperative outcomes.

### Limitations

This report is limited by its single-case design, which restricts the generalizability of the findings and prevents definitive conclusions regarding optimal perioperative management in similar high-risk patients. Although current perioperative and cardiovascular guidelines are well established, individual patients often present unique clinical challenges requiring tailored multidisciplinary decision-making based on the balance between surgical urgency and cardiovascular risk. In our case, management decisions were individualized according to the patient’s urgent surgical indication and complex cardiovascular condition. Future studies involving larger cohorts are needed to better define perioperative anesthetic strategies, hemodynamic management, and optimal timing of urgent noncardiac surgery following recent drug-eluting stent implantation in patients with severe cardiac dysfunction.

### Conclusions

This case illustrates the challenges of perioperative management in a patient with recent coronary stenting, severe multivessel CAD, impaired left ventricular function, and urgent perforated appendicitis requiring emergency surgery. While the management approach was based on established guideline-supported perioperative principles rather than a novel therapeutic strategy, the case highlights the importance of individualized multidisciplinary decision-making when balancing thrombotic, bleeding, and hemodynamic risks in situations where surgical delay is not feasible. The successful integration of continued DAPT, minimally invasive laparoscopic surgery, and advanced hemodynamic monitoring including TEE demonstrates the practical application of these principles in a particularly high-risk clinical setting.

### Learning points

Urgent surgery in recently stented patients is high-risk: early intervention is sometimes unavoidable, but careful balancing of bleeding and thrombotic risk is critical.

Continuation of DAPT is generally protective: maintaining antiplatelet therapy, especially aspirin, reduces the risk of stent thrombosis, even if it may increase perioperative bleeding.

Minimally invasive approaches and low insufflation pressures: laparoscopic techniques with low pneumoperitoneum can limit hemodynamic compromise, reducing perioperative cardiovascular stress.

Physiology-guided anesthesia care: focus on maintaining myocardial oxygen supply–demand balance, avoiding tachycardia, hypotension, or abrupt hypertension, with lung-protective ventilation and careful fluid/vasopressor management.

Multidisciplinary planning and invasive monitoring: coordination among surgery, anesthesia, and cardiology teams, combined with continuous hemodynamic monitoring, enables individualized risk management and optimal perioperative outcomes.

## Data Availability

The authors declare that data supporting the findings of this study are available within the article.
